# Expression and Prognostic Value of Aquaporin 1, 3 in Cervical Carcinoma in Women of Uygur Ethnicity from Xinjiang, China

**DOI:** 10.1371/journal.pone.0098576

**Published:** 2014-06-11

**Authors:** Rui Chen, Yonghua Shi, Reshalaity Amiduo, Talaf Tuokan, Lalai Suzuk

**Affiliations:** 1 Department of Gynecology, Third Affiliated Hospital of Xinjiang Medical University, Urumqi, Xinjiang, China; 2 Department of Pathology, Basic Medicine College of Xinjiang Medical University, Urumqi, Xinjiang, China; State University of Maringá/Universidade Estadual de Maringá, Brazil

## Abstract

**Background:**

Overexpression of several aquaporins has been reported in different types of human cancer but the role of aquaporins in carcinogenesis has not yet been clearly defined. There is few report concerning role of aquaporins in human cervical carcinogenesis so far. Here, we determined the expression and prognostic value of aquaporin 1, 3 in cervical carcinoma in Chinese women of Uygur ethnicity.

**Methods and Results:**

Real-time PCR analyses demonstrated aquaporin 1, 3 mRNA were differentially expressed in cervical carcinoma, CIN 2-3 and mild cervicitis. Immunofluorescent and immunohistochemical analyses demonstrated aquaporin 1 was predominantly localized to stromal endothelial cells in cervical lesions. Aquaporin 3 was localized to the membrane of normal squamous epithelium, CIN and carcinoma cells. Aquaporin 1 and 3 were upregulated in cervical cancer compared to mild cervicitis and CIN2-3 (*P*<0.05); Tumor expression of aquaporin 1, 3 significantly increased in advanced stage disease, and patients with deeper tumor infiltration, lymph node metastases or larger tumor volume (*P*<0.05). Multivariate analysis demonstrated that aquaporin 1, 3 were not independent prognostic factors in cervical carcinoma.

**Conclusion:**

Aquaporins may participate in the initiation and progression of cervical carcinoma by promoting tumor growth, invasion or lymph node metastasis. Further study is required to determine whether aquaporins have potential as prognostic factors in cervical cancer.

## Introduction

Cervical cancer is the third most commonly diagnosed cancer and the fourth leading cause of cancer death in women, accounting for 9% (529,800) of all new cancer cases and 8% (275,100) of all cancer deaths among women in 2008 in the world. More than 85% of these cases and deaths occur in the developing countries, including China [1]. Although early diagnosis and the survival rate for cervical carcinoma have obviously increased in recent years, the efficacy of treatment and prognosis for patients with advanced cervical cancer still remains poor. In Xinjiang, China, especially southern Xinjiang, the incidence of cervical carcinoma in women of Uygur ethnicity is very high, with a prevalence of 527 per 100,000 females; and about 80% of these patients present at an advanced stage [2]. Therefore, further research on the pathogenesis and factors affecting the prognosis of women, especially women of Uygur ethnicity, with cervical carcinoma is of importance.

Aquaporins (AQPs) increase cell plasma membrane water permeability 5–50 times compared with that in membranes where water moves primarily through the lipid bilayer. Since their discovery, 13 mammalian APQ homologs (AQP 0–12) have been identified, which expressed in many epithelia, endothelia, and other types of cells. Phenotype analysis has revealed a variety of important, and in some cases unanticipated physiological roles of AQPs in the urinary concentrating mechanism, glandular fluid secretion, brain swelling, neural excitability, fat metabolism, and skin hydration [3], [4], [5]. Recent studies alluded to the key role of AQPs in human carcinogenesis [6], [7]. For example, AQPs are abnormally expressed in the tumor cells and stroma of malignant tumors in women, such as breast carcinoma and endometrial carcinoma [8], [9]. Human AQP1 expression is observed in gliomas [10]. AQP3 facilitate ovarian cancer cell migration [11]. The role of AQPs in cervical cancer is poorly characterized.

In this study, we investigated the different expression of AQP1andAQP3 in both transcription and translation level in the cervical lesions of women of Uygur ethnicity from Xinjiang, China, and analyzed the prognostic value of AQP1 and AQP3 in cervical carcinoma.

## Materials and Methods

### Ethics Statement

The study was approved by the Ethical Review Committee of Third Affiliated Hospital of Xinjiang Medical University, and the informed consent forms were signed.

### Human Cervical Lesion Tissues

#### 1. Study on setting and population

30 cases of freshly isolated tissues were used to Real-time polymerase chain reaction (RT-PCR) and immunofluorescent analysis. 176 cases of paraformaldehyde-fixed and paraffin-embedded tissues were used for immunohistochemistry, and 98 cases of cervical carcinoma were also used for the prognostic analysis. Clinicopathological information was collected, including age, clinical stage, tumor diameter, pathologic gross type, pathologic grade, tumor infiltration depth, lymphatic metastasis, treatment protocol, serum squamous cell carcinoma-antigen (SCC-Ag) levels, and human papillomavirus (HPV) infection status. Tumor clinical staging was performed according to the Federation International of Gynecology and Obstetrics (FIGO) guidelines [12].

#### 2. Human cervical lesion tissues

All cases were obtained from patients undergoing gynecological surgery at the Department of Gynecology, Third Affiliated Hospital of Xinjiang Medical University. 30 cases of freshly isolated tissues included 10 cases of mild cervicitis, 10 cases of early cervical cancer (stage I + IIA) and 10 cases of advanced cervical cancer. 176 cases of paraffin-embedded tissues included 36 cases of cervical intraepithelial neoplasia (CIN) 2-3, 98 cases of cervical carcinoma and 42 cases of mild cervicitis. The age of the 98 patients with cervical carcinoma ranged from 28–76 years-old (median±standard deviation, 54.28±11.47 years-old). The samples were obtained between November 2007 and August 2010. The inclusion criteria were 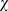 a final pathologic diagnosis of squamous cell carcinoma; 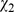 no serious complications; and 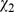 complete pathologic and follow-up data.

### Real-Time PCR

Total RNA was extracted from the freshly isolated human cervical lesion tissues using TRIzol (Invitrogen, Carlsbad, CA, USA), and cDNA synthesis was performed using the First strand cDNA synthesis kit (Promega, Madison, WI, USA) according to the manufacturer’s instructions. RT- PCR was performed using the SYBR green real-time PCR master mix (Fermentas, Lithuania) by pre-denaturalization at 95°C for 5 min; 35 cycles of denaturalization at 95°C for 30 s, annealing at 60°C for 30 s, amplification at 72°C for 30 s and extension at 72°C for 5 min. PCR products were examined by 1.5% agarose gel electrophoresis. The expression of the AQP genes was normalized to the housekeeping gene actin. Primer sequences are shown in Table S1.

### Immunofluorescent Analysis

Tissues were embedded in optimal cutting temperature (OCT; Sakura, Torrance, CA, USA) compound, frozen sections were prepared, fixed for 10 min in cold acetone, the sections were incubated with primary antibodies against AQP1, AQP3 (Santa-Cruz Biotechnology, Santa Cruz, CA, USA) overnight at 4°C, followed by fluorescein isothiocyanate-labeled secondary antibody (Zhongshan Golden Bridge, Beijing, China) for 45 min. After washing, mounting media including buffering glycerine was added, and the coverslips were mounted. PBS was used instead of primary antibody as a negative control; normal cervical tissues were used as AQP-positive controls. The staining was repeated for cases with doubtful staining.

### Immunohistochemistry

Cervical lesion tissues were fixed in 4% paraformaldehyde, embedded in paraffin and 2 µm-thick sections were prepared. The sections were labeled using anti-AQP1, anti-AQP3 primary antibodies (Santa Cruz Biotechnology; at 1∶100, 1∶100, respectively) at 4°C overnight, then incubated with secondary antibody (goat anti-rabbit IgG; Zhongshan Golden Bridge, Beijing, China) for 30 min at room temperature. Following DAB (3,3′-diaminobenzidine) coloration, hematoxylin counterstaining, dehydration and transparency in xylene, the slides were mounted. AQP3 immunolabeling was reviewed and scored by expert pathologists. AQP3 staining intensity was scored as negative (−), weak (score 1), moderate (score 2) or strong (score 3). The proportion of cells stained (the approximate numbers of AQP3- positive tumor cells) was scored as 0–4% (−), 5%–25% (score 1), 26%–50% (score 2), 51%–75% (score 3), or >75% (score 4). The intensity plusing proportion was scored as negative (score 0–1), + (score 2–3), ++ (score 4–5), +++ (score 6–7). Microvessel density (MVD) was used to assess AQP1 immunolabeling. Brown-stained endothelial cell membranes or cytoplasm were considered positive. Three areas of highest neovascularization were found by scanning the sections at low power (100×), and the average MVD value was determined at high power (200×), excluding areas of hemorrhage, inflammatory reaction and borderline areas [13].

### Follow-up

All of 98 cervical cancer patients were followed up with letters and by telephone; the end of follow-up was August 2011. Of the 98 patients, 25 died, and 82 cases were censored. The follow-up time ranged from 7 to 56 months and the 3 year follow-up rate was 85.71%.

### Statistical Analysis

Statistical analysis was performed using SPSS 16.0 (Chicago, IL, USA). Continuous data was expressed as the mean ± standard deviation. Statistical differences among groups were compared using one-way ANOVA for continuous data and the *Χ^2^* test for categorical data. Cumulate survival curves were generated using the Kaplan-Meier method. Cox single-factor analysis using the log-rank test was performed to identify prognostic factors for overall survival. Multivariate analysis was performed using the Cox proportional hazards model. The correlation between AQP expression and survival time was performed using the Spearman’s rank correlation test. *P*<0.05 was considered statistically significant.

## Results

### AQP1 and AQP3 are Associated with Cumulate Survival Rate, but not Independent Risk Factors which Influence the Prognosis of Cervical Carcinoma

**Figure 1:**
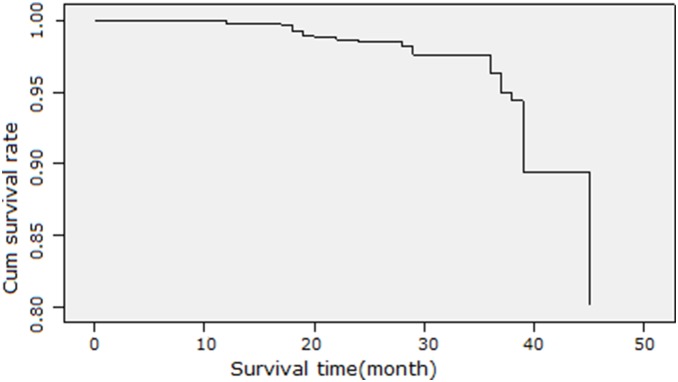
Cumulate survival curve for 98 women of Uygur ethnicity from Xinjiang, China with cervical carcinoma.

**Figure 2:**
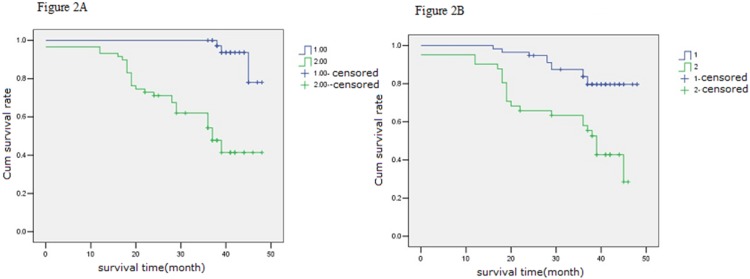
Relationship between AQP expression and cumulate survival curve for 98 women of Uygur ethnicity from Xinjiang, China with cervical carcinoma. A, stratified by expression of AQP1, scored as AQP1 microvessel density (MVD):(1) MVD≤60 (2) MVD>60, P = 0.000; B,stratified by expression of AQP3(1) negative or (2) positive.

The cumulate survival curve for all 98 patients with cervical cancer is shown in Figure 1. The correlations of different clinicopathological features and molecular markers with cumulate survival rate in cervical carcinoma were analyzed using univariate analysis and Kaplan-Meir survival curves. Clinical stage, tumor diameter, lymph node metastasis, tumor infiltration depth, serum SCC-Ag level, HPV infection status, treatment protocol, and expression of AQP1 and AQP3 were associated with cumulate survival rate in cervical cancer (Table 1, Figure 2). Further, multivariate analysis was performed using the Cox proportional hazards model to determine the independent prognostic factors in cervical carcinoma, using the significant variables from the Cox univariate analysis. The only independent significant factors which correlated with cumulate survival were clinical stage, tumor diameter and lymph node metastasis. Tumor infiltration depth, treatment protocol, HPV infection status, serum SCC-Ag level, and AQP1 and AQP3 expression were not independent risk factors in cervical carcinoma (Table 2).

**Table 1:** Cox univariate analysis of cumulate survival for women of Uygur ethnicity with cervical cancer.

Feature	t/ 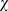 *2* value	*P* value
Age (years)	*t* = 3.576	0.167
Age of first marriage (years)	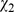 = 0.925	0.336
Age at primiparity (years)	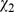 = 2.623	0.144
Gravidity (n)	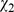 = 0.066	0.797
Childbirth (n)	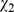 = 0.018	0.894
Number of sexual partners	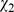 = 0.222	0.638
Clinical stage	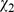 = 41.466	0.000
Tumor diameter	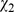 = 14.693	0.000
Pathologic gross type	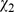 = 1.685	0.09
Pathologic grade	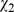 = 1.213	0.545
Lymphatic metastasis	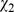 = 34.341	0.000
Infiltration depth	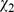 = 31.298	0.000
Serum SCC level	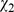 = 5.153	0.023
HPV infection	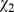 = 3.864	0.048
Treatment protocols	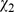 = 22.042	0.000
AQP1	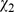 = 25.25	0.000
AQP3	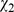 = 14.457	0.000

**Table 2:** Multivariate analysis of prognostic factors in cervical cancer in women of Uygur ethnicity.

Parameter	β	Standard error	WALD 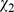	SIG	Exp (β)(OR)	95% CI for Exp (β)	
						Lower	Upper
Clinical stage			20.70	0.037			
Stage I	14.87	325.94	0.002	0.001	1.96	1.00	2.310
Stage II	2.93	0.73	16.25	0.029	1.10	1.053	1.213
Stage III	1.57	0.67	5.58	0.046	1.02	1.00	1.156
Tumor diameter	2.82	0.65	19.13	0.001	1.31	1.060	1.517
Lymphatic metastasis	1.38	0.64	4.74	0.036	1.43	1.25	1.672

Note: using Cox proportional hazards mode.

### Expression of AQP1 and AQP3 mRNA Vary Significantly in Cervical Lesions

**Figure 3:**
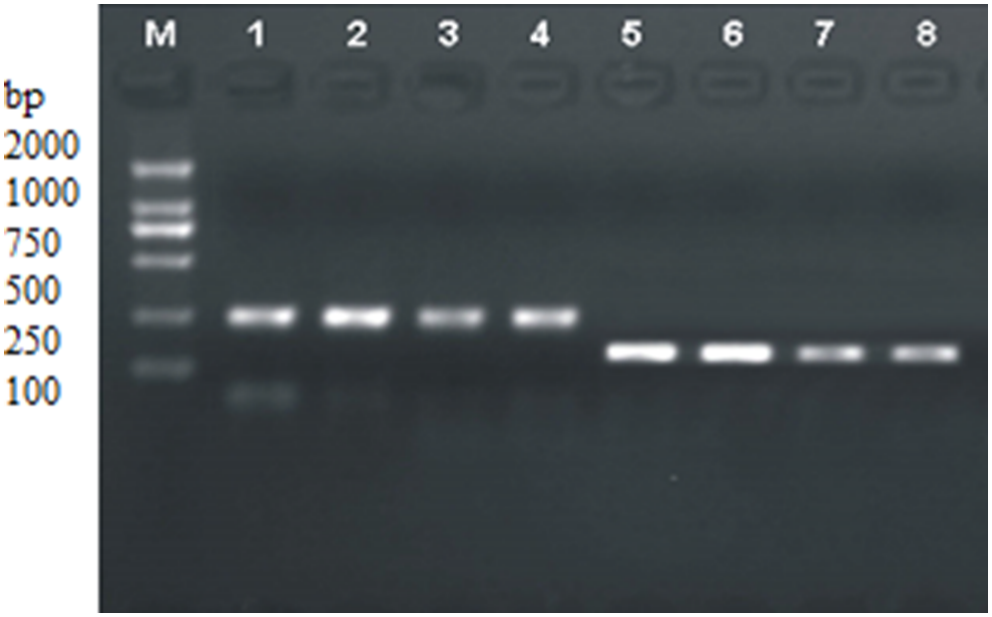
Analysis of AQP1 and AQP3 expression in cervical lesions 1–4: AQP1 RT-PCR electrophotogram; 1and 2, mild cervicitis; 3, early stage cervical cancer; 4, advanced stage cervical cancer. 5–8: AQP3 RT-PCR electrophotogram; 5 and 6, mild cervicitis; 7, early stage cervical cancer, 8, advanced stage cervical cancer.

Real-time PCR was performed to quantify the expression of AQP1 and AQP3 mRNA in different cervical lesions. A representative RT-PCR gel is shown in Figure 3. AQP1 and AQP3 mRNA expression increased from mild cervicitis to cervical carcinoma. The differences in AQP1 and AQP3 mRNA expression between mild cervicitis, early stage and advanced stage cervical carcinoma were significant (*P*<0.05; Table S2).

### Localization and Overexpression of AQP1 and AQP3 in Cervical Lesions

Immunofluorescent and immunohistochemical assays were performed to determine the localization and expression of AQP1 and AQP3 in cervicitis, early stage and advanced stage cervical carcinoma. AQP1 and AQP3 were expressed in mild cervicitis, early stage and advanced stage cervical carcinoma. AQP1 was predominantly localized to the microvascular endothelial cells in the stroma of mild cervicitis, CIN 2-3 and cervical carcinoma (Figure S1A, Figure S2), and the expression of AQP1 was significantly higher in cervical carcinoma than CIN2-3 and mild cervicitis (Figure S4A). AQP3 was localized to the membrane and cytoplasm of normal squamous epithelium and carcinoma cells, showed intense staining in cervical cancer (Figure S1B, Figure S3). Positive expression rate of AQP3 gradually increased from mild cervicitis and CIN2-3 to cervical cancer (*P*<0.05) (Figure S4B).

### Expression of AQP1 and AQP3 is Related to Clinical Stage, Tumor Diameter, Lymphatic Metastasis and Tumor Infiltration Depth in Cervical Carcinoma

One-way ANOVA, the *Χ^2^* test and rank correlation analysis demonstrated that positive expression rates of AQP1 and AQP3 were significantly related to clinical stage, tumor diameter, lymphatic metastasis and the tumor infiltration depth in cervical carcinoma (Table S3, S4).

## Discussion

AQPs are small transmembrane proteins that facilitate osmotically driven water transport. All vital processes in cancerous cells depend on water in the tumor microenvironment, and tumor cells require enhanced water transmembrane transport compared to normal cells. AQPs are overexpressed in different cancers, as well as the vascular endothelial cell lines and tumor cell lines derived from tumors, suggesting that AQPs are closely associated with tumorigenesis and tumor progression. For example, AQP5 overexpression is related to cell growth and metastasis in human breast cancer [14]. AQP3 and AQP5 are upregulated in gastric carcinoma, and are associated with lymph node metastasis and lymphovascular invasion [15]. The expression of AQP1, AQP5 and AQP9 are significantly higher in malignant and borderline ovarian tumors than benign tumors and normal ovarian tissues [16]. However, the expression and role of AQPs in human cervical carcinogenesis are poorly characterized.

In our previous study, we observed that AQP1and AQP3 are the only members of the AQPs family to be overexpressed in cervical cancer [17]. In this study, we tested AQP1 and AQP3 expression in cervical lesion tissues by RT-PCR, and immunofluorescent and immunohistochemical analysis. We also analyzed the correlation between AQP1 and AQP3 expression and prognosis in cervical carcinoma. We observed that AQP1 and AQP3 exhibited different expression patterns in cervical carcinoma, CIN and normal tissues at both the mRNA and protein levels. Immunohistochemical and immunofluorescent assays demonstrated that AQP1 was mainly expressed in vascular endothelial cells; whereas AQP3 was localized to the epithelial cellular membrane in cervical lesions. MVD is an accepted, reliable indicator of tumor angiogenesis [18]. In this study, MVD was used to assess the expression of AQP1 protein, as it was localized to the tumor microvessels. Expression of AQP1 and AQP3 were remarkably upregulated in cervical carcinoma tissues compared to CIN and mild cervicitis. These results indicate that these AQPs may be related to cervical carcinogenesis and the progression of cervical cancer.

We also analyzed a number of clinicopathologic and molecular factors which may influence prognosis in cervical carcinoma. Cox univariate analysis demonstrated that advanced clinical stage, large tumor diameter, lymphatic metastasis, increased tumor infiltration depth, as well as molecular markers such as an abnormal serum SCC-Ag level, HPV infection and overexpression of AQP1, AQP3 were significantly related to poorer overall survival in cervical carcinoma. However, multivariate analysis indicated that overexpression of AQP1and AQP3 were not independent risk factors associated with prognosis in cervical cancer.

To further investigate how AQPs may affect prognosis in cervical carcinoma, the relationships between AQP expression and the clinicopathological parameters of cervical carcinoma were analyzed. Tumor expression of AQP1 and AQP3 were significantly higher in advanced stage disease, patients with metastatic lymph nodes, a larger tumor size or deeper tumor infiltration, suggesting that these AQPs may influence the prognosis of cervical cancer by promoting tumor growth, invasion and lymphatic metastasis.

Our results indicate that AQP1 and AQP3 are closely associated with tumor vascularization, the progression, invasion and metastasis of cervical carcinoma. There are no blood vessels during the initial phase of tumor growth, when the tumor tissues mainly acquire nutrition via interstitial fluid diffusion. When a tumor reaches a radius of 1–2 mm, angiogenesis is required to enable continued growth of the tumor. Formation of neoplastic metastatic foci also requires the process of angiogenesis. In *AQP1*-knockout mice, xenograft tumor growth and angiogenesis were reduced, and significant necrosis occurred in the tumor tissues [19]. Kao et al. [20] reported that the expression of AQP1 correlated significantly with prognosis in malignant mesothelioma, irrespective of treatment or established prognostic factors. Kusayama et al. [21] observed high level expression of AQP3 in primary squamous cell carcinomas such as esophageal and lingual cancers, as well as the corresponding lymphatic metastases. Overexpression of AQP3 was observed on tumor cells in oral squamous carcinoma [22]. AQPs participate in body water homeostasis, we propose that water metabolism through AQP1 and AQP3 is maintained during neoplastic transformation in human cervical tissues. AQPs overexpression in cervical cancer may increase tumor cells permeability to water to alter tumor cells volume and shape, accordingly facilitate advancement, infiltration and metastasis of cervical carcinoma.

In summary, AQP1and AQP3 are upregulated in cervical carcinoma in women of Uygur ethnicity from Xinjiang, China. Upregulation of AQP1 and AQP3 may facilitate progression, invasion and metastasis in cervical carcinoma, suggesting that AQPs may represent potential targets for the treatment of cervical carcinoma in the future. However, further studies are required to determine the ability of AQPs to function as molecular markers for predicting prognosis in cervical cancer.

## Supporting Information

Figure S1 **Immunofluorescent analysis of AQP1 and AQP3 localization in cervical carcinoma.** A, AQP1 is expressed in microvascular endothelial cell of cervical carcinoma. B, AQP3 is expressed in the membrane of cervical carcinoma cells (×400).
(TIF)

Figure S2 **Immunohistochemical analysis of AQP1 expression in cervical lesions.** A, AQP1 expression in microvascular endothelium of cervical cancer. B, AQP1 expression in CIN. C, AQP1 expression in mild cervicitis. D, Negative control of AQP1 expression in cervical cancer (×100).
(TIF)

Figure S3 **Immunohistochemistry figure of AQP3 expression in cervical lesions.** A, AQP3 diffuse expression in cervical cancer. B, AQP3 expression in CIN. C, AQP3 expression in mild cervicitis. D, Negative control of AQP3 expression in cervical cancer (×100).
(TIF)

Figure S4 **Immunohistochemiscal analysis of AQP1, 3 expression in cervical lesions.** A, AQP1(MVD) differential expression in cervical lesions, *P<0.05 vs CIN2-3, **P<0.01 vs cervical cancer, ***P<0.01 vs mild cervicitis, B, AQP3 differential expression in cervical lesions, *P<0.01.
(TIF)

Table S1 **Primer sequences and size of the AQP1, AQP3 and housekeeping gene actin PCR products.**
(DOCX)

Table S2
**Expression of AQP1 and AQP3 mRNA in cervical lesions (x±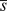).** Note: using one-way ANOVA analysis. *: Didderent expression of AQP1 mRNA among mild cervicitis, early cervical cancer and advanced cervical cancer; **: Didderent expression of AQP3 mRNA among mild cervicitis, early cervical cancer and advanced cervical cancer.(DOCX)

Table S3 **Relationship of AQP1 and AQP3 protein expression with the clinicopathologic features of cervical carcinoma.**
(DOC)

Table S4 **Correlation analysis of AQP1, AQP3 protein expression with clinicopathologic parameters in cervical carcinoma.** Note: using Spearman’s rank correlation test.
(DOCX)
