# Supplementary figures and images for: Expression and Prognostic Value of Aquaporin 1, 3 in Cervical Carcinoma in Women of Uygur Ethnicity from Xinjiang, China
Source: PLoS One. 2014 Jun 11;9(6):e98576. doi: 10.1371/journal.pone.0098576 (PMC4053468; doi:10.1371/journal.pone.0098576)

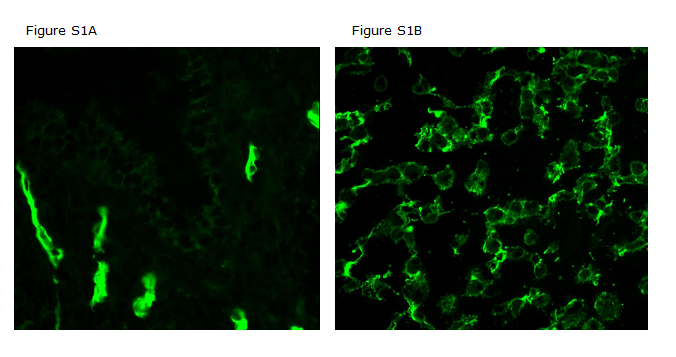

Supplement: Figure S1 — Immunofluorescent analysis of AQP1 and AQP3 localization in cervical carcinoma. A, AQP1 is expressed in microvascular endothelial cell of cervical carcinoma. B, AQP3 is expressed in the membrane of cervical carcinoma cells (×400). (TIF) [file pone.0098576.s001.tif]

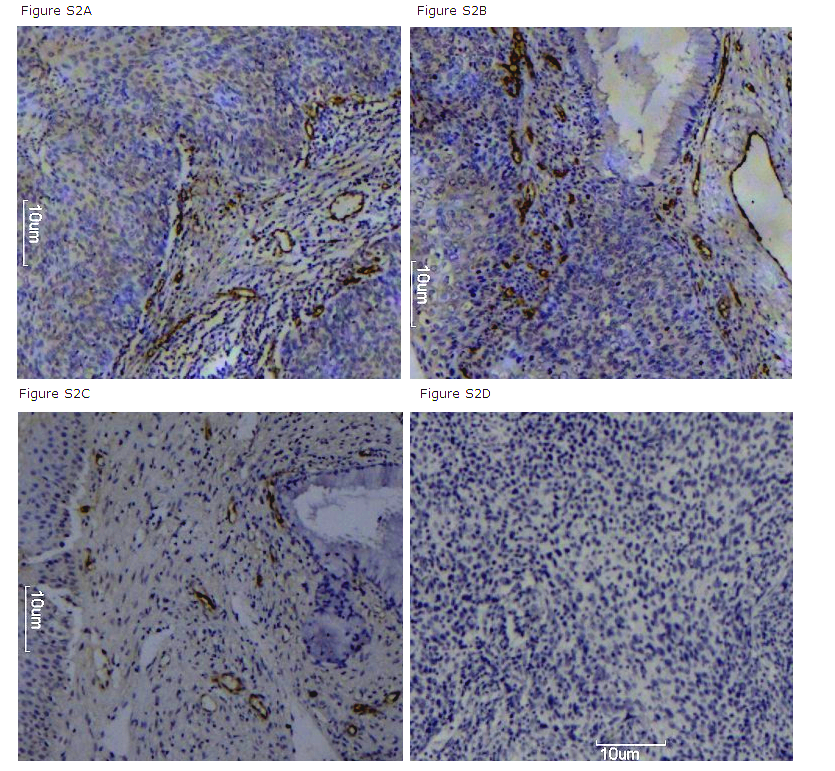

Supplement: Figure S2 — Immunohistochemical analysis of AQP1 expression in cervical lesions. A, AQP1 expression in microvascular endothelium of cervical cancer. B, AQP1 expression in CIN. C, AQP1 expression in mild cervicitis. D, Negative control of AQP1 expression in cervical cancer (×100). (TIF) [file pone.0098576.s002.tif]

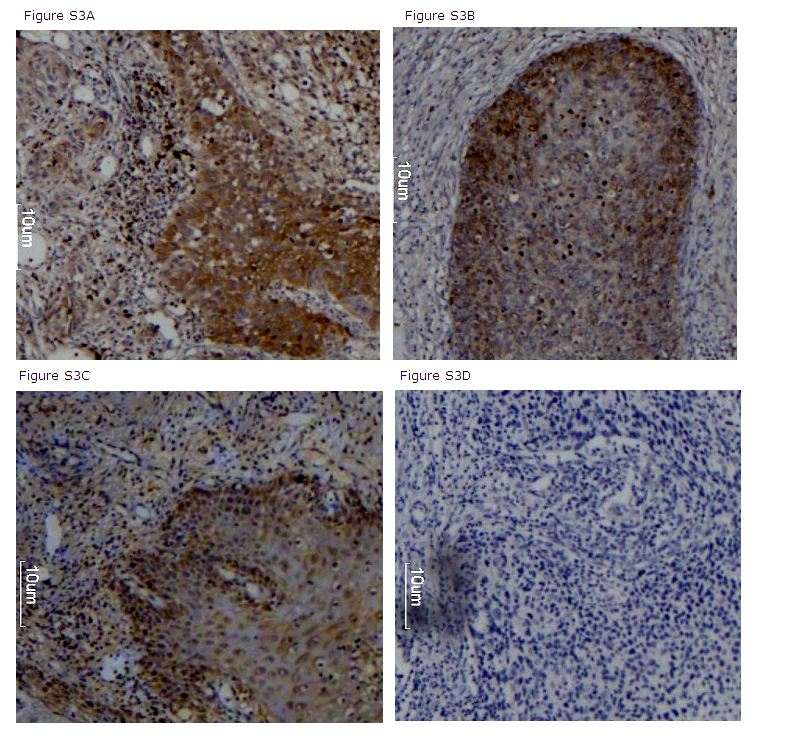

Supplement: Figure S3 — Immunohistochemistry figure of AQP3 expression in cervical lesions. A, AQP3 diffuse expression in cervical cancer. B, AQP3 expression in CIN. C, AQP3 expression in mild cervicitis. D, Negative control of AQP3 expression in cervical cancer (×100). (TIF) [file pone.0098576.s003.tif]

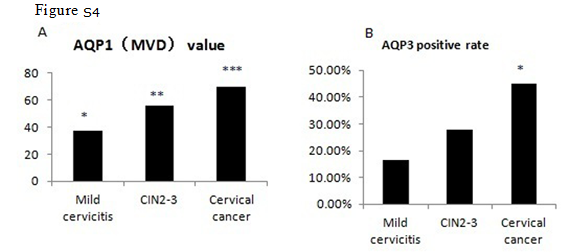

Supplement: Figure S4 — Immunohistochemiscal analysis of AQP1, 3 expression in cervical lesions. A, AQP1(MVD) differential expression in cervical lesions, *P<0.05 vs CIN2-3, **P<0.01 vs cervical cancer, ***P<0.01 vs mild cervicitis, B, AQP3 differential expression in cervical lesions, *P<0.01. (TIF) [file pone.0098576.s004.tif]
