# Supplementary material for: Expression and Prognostic Value of Aquaporin 1, 3 in Cervical Carcinoma in Women of Uygur Ethnicity from Xinjiang, China
Source: PLoS One. 2014 Jun 11;9(6):e98576. doi: 10.1371/journal.pone.0098576 (PMC4053468; doi:10.1371/journal.pone.0098576)
Supplement: Table S1 — Primer sequences and size of the AQP1, AQP3 and housekeeping gene actin PCR products. (DOCX) [file pone.0098576.s005.docx]

| **Table S1. Primer sequences and size of the AQP1, AQP3 and housekeeping gene actin PCR products** | | |
| --- | --- | --- |
| Primer | Sequence | Size of product |
| AQP1 sense | GCTGGTGCTATGCGTGCTG | 250 bp |
| AQP1 antisense | CAGGATGAAGTCGTAGATGAGTACAG |  |
| AQP3 sense | CATTGCGGGTGTCTTCGTG | 177 bp |
| AQP3 antisense | GGACAGTCAGTGGATGCTCAAG |  |
| Actin sense | CATGTACGTTGCTATCCAGGC | 250 bp |
| Actin antisense | CTCCTTAATGTCACGCACGAT |  |
