# Supplementary material for: Expression and Prognostic Value of Aquaporin 1, 3 in Cervical Carcinoma in Women of Uygur Ethnicity from Xinjiang, China
Source: PLoS One. 2014 Jun 11;9(6):e98576. doi: 10.1371/journal.pone.0098576 (PMC4053468; doi:10.1371/journal.pone.0098576)
Supplement: Table S2 — Expression of AQP1 and AQP3 mRNA in cervical lesions (x±). Note: using one-way ANOVA analysis. *: Didderent expression of AQP1 mRNA among mild cervicitis, early cervical cancer and advanced cervical cancer; **: Didderent expression of AQP3 mRNA among mild cervicitis, early cervical cancer and advanced cervical cancer. (DOCX) [file pone.0098576.s006.docx]

| **Table S2. Expression of *AQP1* and *AQP3* mRNA in cervical lesions (x ±$\overline{s}$)** | | | | | |
| --- | --- | --- | --- | --- | --- |
| AQP | Group | *n* | Expression abundance | *F* value | *P* value |
| *AQP1* | Mild cervicitis | 10 | 1.007±0.016 | 210.9 | < 0.01* |
|  | Early cervical cancer | 10 | 1.770±0.006 |  |  |
|  | Advanced cervical cancer | 10 | 3.954±0.262 |  |  |
| *AQP3* | Mild cervicitis | 10 | 1.008±0.019 | 227.5 | < 0.01** |
|  | Early cervical cancer | 10 | 1.999±0.064 |  |  |
|  | Advanced cervical cancer | 10 | 3.721±0.166 |  |  |

Note: using one-way ANOVA analysis

*: Different expression of AQP1 mRNA among mild cervicitis, early cervical cancer and advanced cervical cancer

**: Different expression of AQP3 mRNA among mild cervicitis, early cervical cancer and advanced cervical cancer
