# Supplementary material for: Expression and Prognostic Value of Aquaporin 1, 3 in Cervical Carcinoma in Women of Uygur Ethnicity from Xinjiang, China
Source: PLoS One. 2014 Jun 11;9(6):e98576. doi: 10.1371/journal.pone.0098576 (PMC4053468; doi:10.1371/journal.pone.0098576)
Supplement: Table S3 — Relationship of AQP1 and AQP3 protein expression with the clinicopathologic features of cervical carcinoma. (DOC) [file pone.0098576.s007.doc]

|  | | |
| --- | --- | --- |
|  |  |  |
|  |  |  |
|  |  |
|  |  |  |
|  |  |
|  |  |  |
|  |  |

|  | | | | | |
| --- | --- | --- | --- | --- | --- |
|  |  |  |  |  |  |
|  |  |  |  |  |  |
|  |  |  |  |  |
|  |  |  |  |  |
|  |  |  |  |  |  |
|  |  |  |  |  |
|  |  |  |  |  |

**Table S3. Relationship of AQP1 and AQP3 protein expression with the clinicopathologic features of cervical carcinoma**

| Clinico- | n | AQP1 | | *P* value | AQP3 | | |
| --- | --- | --- | --- | --- | --- | --- | --- |
| pathologic parameter |  | MVD | F value |  | + | х^2^ value | *P* value |
| Stage |  |  |  |  |  |  |  |
| Ⅰ | 14 | 59.83±18.56 | 6.633 | 0.002* | 4 | 6.39 | 0.048** |
| Ⅱ | 52 | 68.53±21.59 |  |  | 20 |  |  |
| Ⅲ | 32 | 79.87±19.89 |  |  | 20 |  |  |
| Infiltrating deepth |  |  |  |  |  |  |  |
| ≤1/2 | 55 | 68.71±23.77 | 6.76 | 0.011 | 19 | 4.28 | 0.043 |
| ﹥1/2 | 43 | 72.07±20.86 |  |  | 25 |  |  |
| Metastatic lymph node |  |  |  |  |  |  |  |
| - | 42 | 65.01±21.94 | 12.038 | 0.001 | 20 | 12.513 | 0.000 |
| + | 56 | 78.16±14.23 |  |  | 24 |  |  |
| Tumor diameter |  |  |  |  |  |  |  |
| ≤5cm | 40 | 66.41±19.12 | 5.987 | 0.001 | 14 | 16.45 | 0.000 |
| ＞5cm | 58 | 76.28±23.10 |  |  | 30 |  |  |

|  |  |  |  |  |
| --- | --- | --- | --- | --- |
|
|  |  |  |  |  |
|  |  |  |  |  |
|  |  |  |  |  |
|  |  |  |  |  |
|  |  |  |  |  |
