# Supplementary material for: Expression and Prognostic Value of Aquaporin 1, 3 in Cervical Carcinoma in Women of Uygur Ethnicity from Xinjiang, China
Source: PLoS One. 2014 Jun 11;9(6):e98576. doi: 10.1371/journal.pone.0098576 (PMC4053468; doi:10.1371/journal.pone.0098576)
Supplement: Table S4 — Correlation analysis of AQP1, AQP3 protein expression with clinicopathologic parameters in cervical carcinoma. Note: using Spearman’s rank correlation test. (DOCX) [file pone.0098576.s008.docx]

**Table S4. Correlation analysis of AQP1, AQP3 protein expression with clinicopathologic parameters in cervical carcinoma**

| Feature | AQP1 |  | AQP3 |  |
| --- | --- | --- | --- | --- |
|  |  |  |  |  |
|  | r | *P* | r | *P* |
| Clinical stage | 0.574 | 0.000 | 0.251 | 0.038 |
| Tumor diameter | 0.251 | 0.013 | 0.426 | 0.000 |
| Metastatic lymph nodes | 0.457 | 0.000 | 0.357 | 0.000 |
| Infiltration depth | 0.358 | 0.000 | 0.209 | 0.039 |

Note: using Spearman’s rank correlation test
